# Supplementary material for: Different cell imaging methods did not significantly improve immune cell image classification performance
Source: PLoS One. 2022 Jan 27;17(1):e0262397. doi: 10.1371/journal.pone.0262397 (PMC8794178; doi:10.1371/journal.pone.0262397)
Supplement: S1 File — (DOCX) [file pone.0262397.s001.docx]

**Supporting information**

**S1 Table. Number of cells in Experiment 2**

|  | **Experiment 2** |
| --- | --- |
| **Number of cells cropped** | 56,644 |
| **Number of cells labelled** | 51,191 |
| **Number of cells used for learning** | 46,280 |

For Experiment 2, one shot of Alx594 and 30 continuous shots (3 s) of BF, DIC and Ph at the same focal position were recorded sequentially and repeated for 204 fields of view. The same procedure was performed on three different observation dishes. Labelling was performed in the same way as described for Experiment 1. The range of unused was −2.468 < *F_i_* < −2.188.

**S1 Fig. Data for Experiment 2.** (A, B) Cross-validation results for CNN inputs in Experiment 2. Triangles, circles and squares represent validations 1, 2 and 3, respectively. In Experiment 2, the same level of AUC could be achieved when using CNN. However, identification by pixel number or contour showed higher identification performance than in Experiment 1 because in Experiment 2 the size histogram had less overlap by cell type. (C) Size histograms for LMPP and pro-B cells. Brown represents LMPP and green represents pro-B cells in both experiments. The Experiment 2 histogram had less overlap by cell type. (D) Intensity distribution of Alx594-conjugated anti-CD19 antibody fluorescence in Experiment 2 colour-coded by LMPP, pro-B and unused.


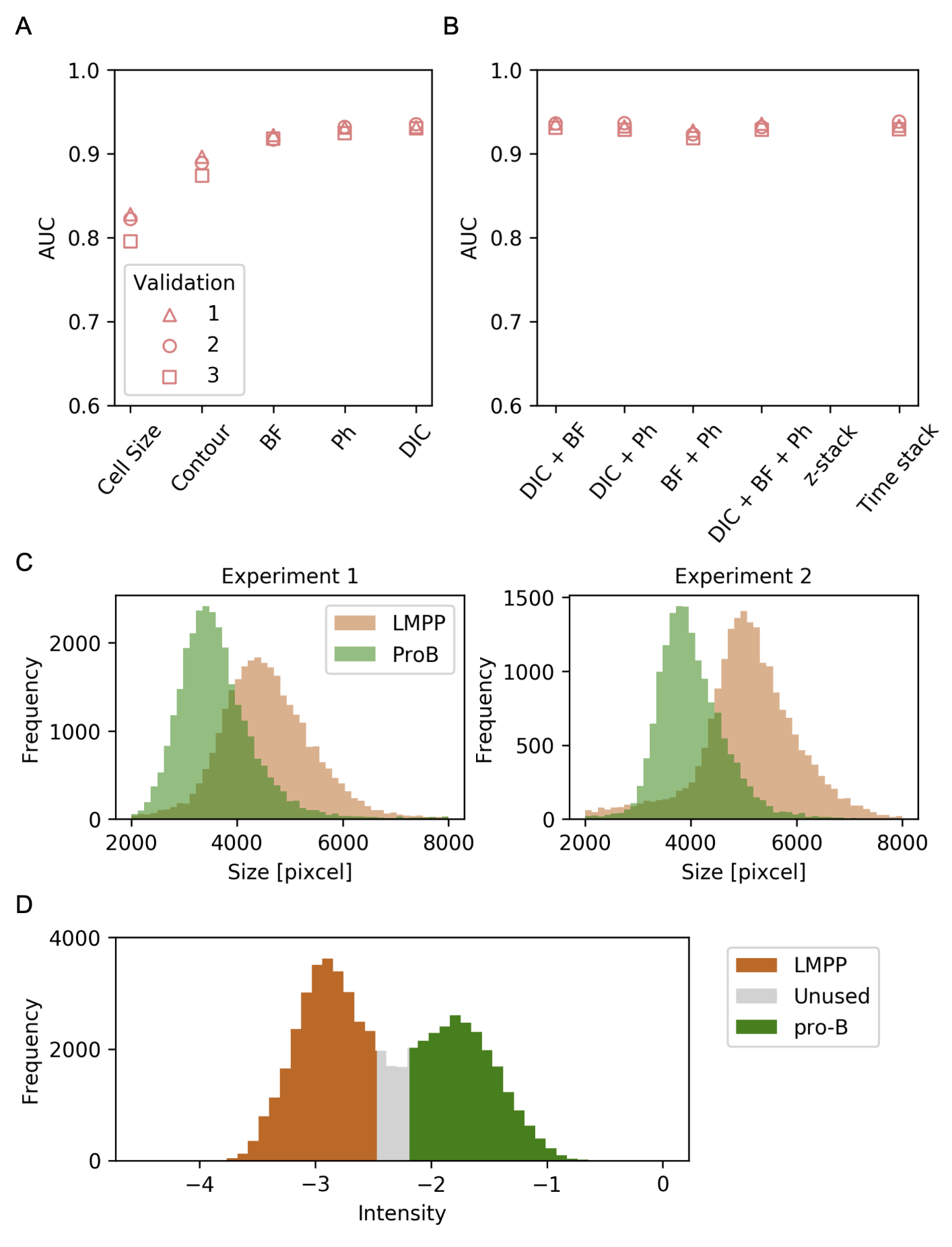


**S2 Fig. AUC related to the focal position for BF and Ph.** Effects of focus shift on AUC in BF images and Ph images.


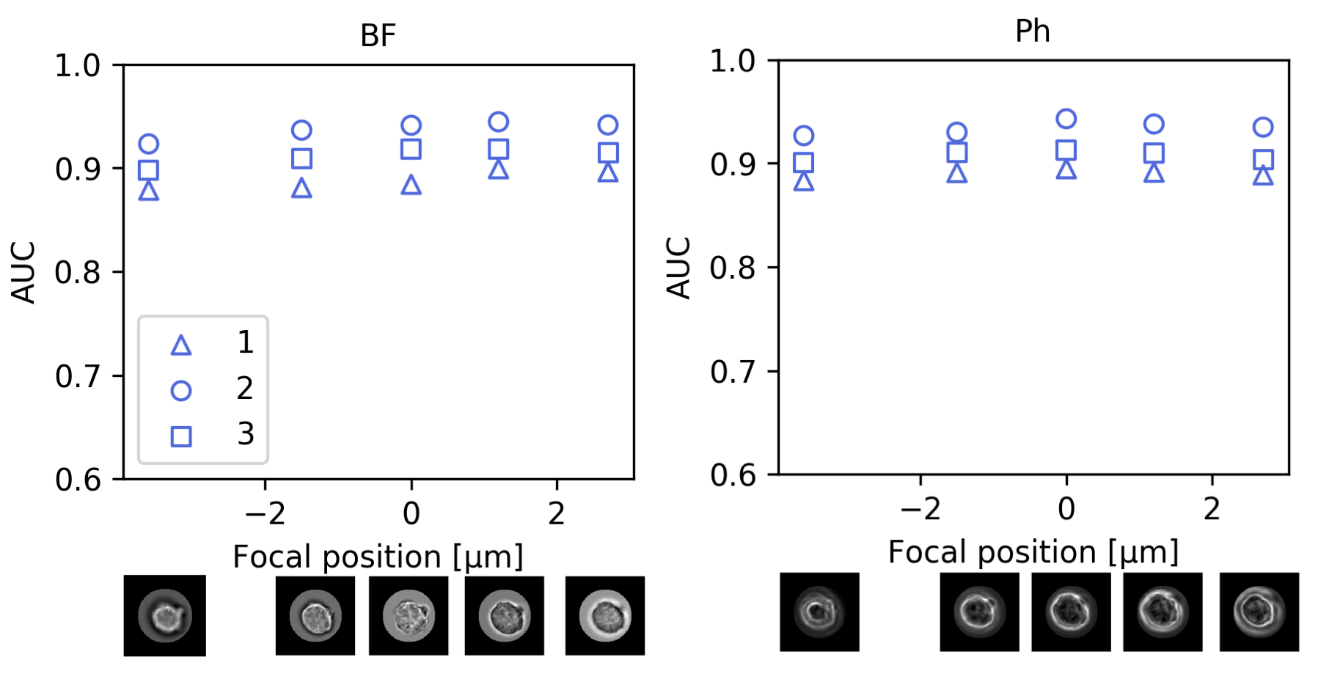


**Dataset**

As a minimum data set, 88 images (4 channels (Alx594-conjugated anti-CD19 antibody fluorescence, BF, Ph, DIC) × 22 focal positions) were extracted from Experiment 1, and 91 images (3 channels (BF, Ph, DIC) × 30 time step + one Alx594-conjugated anti-CD19 antibody fluorescence) from Experiment 2. Since the images were extracted by dish, a total of 537 images (3 dish × 88 images for Experiment 1 and 3 dish × 91 images for Experiment 2) were published on Figshare This is about 0.6% of the total number of images.
