# Supplementary material for: Different cell imaging methods did not significantly improve immune cell image classification performance
Source: PLoS One. 2022 Jan 27;17(1):e0262397. doi: 10.1371/journal.pone.0262397 (PMC8794178; doi:10.1371/journal.pone.0262397)
Supplement: Attachment — Submitted filename: Responses to Reviewers-rev.docx [file pone.0262397.s002.docx]

# **Responses to Reviewers’ Concerns for Taisaku Ogawa et al., “Different cell imaging methods did not significantly improve immune cell image classification performance”**

We would like to thank all the reviewers for their insightful, critical, and constructive comments for improving the manuscript. We are encouraged by the overall positive and enthusiastic responses from all reviewers.

According to the reviewer's comments, we have further refined our manuscript by clarifying descriptions. We are confident that we have addressed all of the reviewers’ concerns by incorporating reviewers’ suggestions into the revised manuscript. We also believe that it now better conforms to the publication guidelines of PLOS ONE.

Here is the summary of the updates; First, we added further clarifications or modified some parts of the manuscript. Second, we responded to Reviewer #2’s concerns on focal positions by conducting some additional analyses. Third, we addressed a number of minor flaws kindly pointed out by the reviewers to improve clarity. All changes we made are highlighted in yellow in the revised manuscript.

## Reviewer #1

### Concern #1

I do not find enough merit to support the publication of the paper, as the contribution is not significant enough. The authors do not compare the proposed approach with actual state of art. Several methods are proposed in the literature. It is not clear the novelty of the method. Are not clear the pros and cons of the proposed approach respect the actual state of the art. Overall impression of study is just below average; the study could be useful and deals with significant problem, but the style way in which paper is written does not match the journal standards.

The purpose of this study is not to propose novel methods nor to demonstrate performance over the state of the art. We investigated the impact of using different observation methods on cell classification accuracy. The impact of cell observation methods on cell type identification has not been systematically investigated, and we believe that our work conveys useful information for a broad range of experimentalists and data scientists working in the area of cell imaging. We have made this clear in the introduction (Main text lines 49-63).

## Reviewer #2

### Concern #1

The authors should include a clear description of experimental and observational factors and their levels in this experimental design study. Right now, the reader cannot easily derive such critical information to this study. For example, it is not clear whether the cells are going through mitosis during time lapse imaging and therefore the cell state is ignored as a factor.The focal position is listed as a factor. The levels of this factor are captured as z-stacks (± 3.0 μm range, 0.3-μm interval). First, the authors should explain how they samples the focal positions (e.g., frequently occurring deviation from a perfect focus, visually indistinguishable image appearance). Second, the claim about classification accuracy being invariant to focal position should be limited to the chosen range (otherwise it might not be true).

*The authors should include a clear description of experimental and observational factors and their levels in this experimental design study. Right now, the reader cannot easily derive such critical information to this study.*

We thank the reviewer’s comments. We modified the main text accordingly and explained details for the two comments below.

*For example, it is not clear whether the cells are going through mitosis during time lapse imaging and therefore the cell state is ignored as a factor.*

Our data included the cells which were going through mitosis. We intended to classify differentiation states without distinction of cell cycle. We modified the main text accordingly (line 94-96).

*The focal position is listed as a factor. The levels of this factor are captured as z-stacks (± 3.0 μm range, 0.3-μm interval). First, the authors should explain how they samples the focal positions (e.g., frequently occurring deviation from a perfect focus, visually indistinguishable image appearance). Second, the claim about classification accuracy being invariant to focal position should be limited to the chosen range (otherwise it might not be true).*

To explain how we determined the focal positions, we added the following description in the main text. We also performed analyses below which showed reproducibility of the home focal position (0 µm in Z-stacks) determination in different field of view by a perfect focus system. Based on the analyses, we keep the statement in the main text (line 258-260), “Cross-validation while shifting the focus on DIC images resulted in no significant degradation of the AUC in the range examined (from - 3.6 µm to + 2.7 µm) (Fig 4C). The same results were found for BF and Ph (S2 Fig)”.

Main text line 110-118:

“The home focal position (0 µm in Z-stacks) was determined by observation of 56 cells in the first field of view of the first dish; a focal position where the area of most cells in Alx594-conjugated anti-CD19 antibody fluorescence images were apparently (by eyes) the largest was defined as the home focal position. This home focal position was recorded using Nikon Perfect Focus System (PFS) (TI2-N-ND-P), and used for all three dishes in Experiment 1. Focal position was initially moved to the home focal position before taking Z-stacks of each channel using PFS, and subsequently moved from from - 3.6 µm to + 2.7 µm with 0.3 µm interval by the motorized focusing unit of Ti2-E microscope.”

Main text line 121-123:

“The focal position was determined and recorded as Experiment 1. The focal position was moved to the home focal position before taking each shot of each channel using PFS.”

First, we showed images of some cells in the Alx594-conjugated anti-CD19 antibody fluorescence channel in the first field of view from the first dish and also histograms of the pixel intensity values of the Alx594-conjugated anti-CD19 antibody fluorescence of the first field of view from the first dish for different focal positions (Fig. R1A, B). We found that the standard deviation of the histogram changed as a function of the focal positions and had the maximum value (Z_max_) around the home focal position (0 µm) (Fig. R1C). Therefore, we calculated the standard deviation of the pixel intensity values at each focal position of all field of view (dish1, 2, and 3) and fitted a Gaussian distribution to the standard deviations between Z_max_ - 1.5 µm and Z_max_ + 1.5 µm (Fig. R1C, R2A). The standard deviation of the peaks of the Gaussian curves were 0.38 μm (Fig. R2B), which is supposed to correspond to the error range of the home focal positions in our experiments. This value is significantly smaller than the focal position range, 6.3 μm (from - 3.6 µm to + 2.7 µm), and is comparable with the interval (0.3μm).

**Fig R1.** (A) Z-stacks of the Alx594-conjugated anti-CD19 antibody fluorescence image at the first field of view of the first dish in Experiment 1. Numbers indicate the focal positions. Scale bar, 20 µm. This is one part of the field of view. (B) Histogram of the pixel intensity values of the Alx594-conjugated anti-CD19 antibody fluorescence image at different focal positions (-2.4, -1.2, 0, 1.2, 2.4 µm) in the first field of view of the first dish in Experiment 1. (C) Standard deviation of the pixel intensity depending on the focal positions in the first field of view of the first dish in Experiment 1. Dots; calculated standard deviation, Lines; fitted curve.

**Fig R**2 (A) Normalized standard deviation of the pixel intensity depending on the focal positions in Experiment 1. The values for each field of view were normalized to the range of 0 to 1. Dots; calculated standard deviation after normalization, Colors; different fields of view, Lines; the fitted curves. (B) Distribution of position of the fitted peak. The mean value was -0.54 µm and the standard deviation was 0.38 µm (four fields of view were not included in A and B because there was a large bright cluster possibly due to a dust).

### Concern #2

The authors should explain why they used a custom designed CNN architecture as opposed to using a widely used U-Net CNN architecture. The authors could include a picture of the custom-designed CNN architecture. If the authors are not using the U-Net, then they could compare the CNN architectural designs (custom vs U-Net)

Our CNN network is a plain CNN (no special connections such as skip connections and attention are added), and only changes made were on network parameters such as kernel sizes. Parameters used are given in the section ‘Training and testing of CNN and SVM’ (Main text lines 210-213).

As far as we understand, U-Net is a network for image transformation and is not often used for identification. Although the main focus of this study is to investigate the effect of different observation methods on the accuracy of identification, it is an interesting question whether our findings hold for other architectures (such as ResNet). We have added this point to Discussion (Main text lines 296-302).

### Concern #3

The authors should include confidence intervals in their reported results. For example, “CNN showed the best classification performance with DIC images (AUC ~0.9).” on page 15. How do we know that the classification results obtained using DIC images are statistically any better than the results obtained by the other imaging modalities?

There is no clear criteria to say that DIC is better than others. The relevant description has been deleted (Main text line 242).

### Concern #4

Page 8: “These regions were removed from the train/test data by removing regions ≤ 2000 pixels and ≥ 8000 pixels”. Can you provide physical dimensions accompanied by some apriori knowledge?

For all microscopic images in this study, 162.5 nm/pix was used, so 2000 pixels and 8000 pixels correspond to 52.81 µm^2^ and 211.2 µm^2^ , respectively. A description of unit conversion has been added to the Materials and methods section (Main text lines 142-144).

### Concern #5

Page 9: “…fluorescence images were divided by the intensity of the blurred Alx594-conjugated ..” Who defines which frame in a z-stack is blurred?

In order to remove the effect of background luminance, a gaussian filter with sigma=200[pixel] was applied to each microscope image to produce a blurred image, as I added in main text lines 157-158. The value of sigma was empirically determined by the author while viewing the images after background removal.

### Concern #6

Page 11: “Cell images were masked with a circle of radius 50 pixels …” Why are the cell images masked with a circle if you have a segmentation mask? I would also recommend clarify the terminology for raw cell images, mask images from segmentation (ground truth), and mask images created by inserted circles into background color.

The mask was generated from the Phase contrast image. When this mask is applied, the information from the phase contrast affects the images in the other channels through the shape. To avoid this, we applied a circular mask to the images used for training and evaluation. We did not choose to use the no mask method because without it, other cells on the edges could affect the evaluation. Explanation added to the main text (lines 196-198).

### Concern #7

Page 16, line 258: “different numbers of cell images (214, 708, 2,144, 7,077 and 21,444 cells)” Please, use semicolons to separate the numbers if you are using commas to separate thousands. Include the numbers of cell images for CNN training into a list of factors explored.

Done (Main text lines 264-265).

### Concern #8

The authors claim that the data are available, but there is no URL pointing to the data.

The data will be made available from the corresponding author upon reasonable request.

Since the size of the datasets is larger than 1 TB, it is difficult to put it on a public place with a URL.

## Reviewer #3

### Concern #1

Hence, it is not clear if the reported findings represent a general theme or restricted to this very system that the authors demonstrated in the study. As it is well known that the performance of deep learning is highly associated with network architectures. Thus, it is not clear if or how different network architecture (such as ResNet, google net, mobile net …etc.) of deep learning can affect the classification performance in recognizing different types of biological specimens/cell samples under different imaging modalities. Overall, more thorough studies should be conducted to better support their claims.

As pointed out by reviewer #3, we conducted our analyses on some specific cell types (LMPP and pro-B cells), and used a particular type of network architecture (CNN). We agree that more thorough studies should be conducted to better understand the dependence of cell classification accuracy on different types of cell samples, imaging modalities, and network architectures, exactly as we stated in the last sentence of Discussion. However, we do not think that our claim must be tested on all cell types nor all possible network architectures (there are hundreds!), because it is not what we would like to show in this study. Our claim is that under properly designed experimental and analyses setup (such as image processing workflow and the number of images taken; see e.g. Fig2, Fig 4D), there is at least one network architecture which attains acceptable classification accuracy (i.e. AUC >~ 0.9), regardless of some important factors including imaging modalities and focal position, which sometimes be considered suspected affecters. We showed that if a suitable network architecture is chosen and imaging experiments were properly conducted, it is possible to get rid of the effect of these factors. We think that this finding is useful to experimentalists and data scientists working in cell imaging in choosing their imaging modalities and designing data processing workflow.

We further emphasized this in Discussion (Main text lines 296-302).
