# Supplementary material for: Different cell imaging methods did not significantly improve immune cell image classification performance
Source: PLoS One. 2022 Jan 27;17(1):e0262397. doi: 10.1371/journal.pone.0262397 (PMC8794178; doi:10.1371/journal.pone.0262397)
Supplement: Attachment — Submitted filename: Responses to Reviewers.docx [file pone.0262397.s003.docx]

# **Responses to Reviewers’ Concerns for Taisaku Ogawa et al., “Different cell imaging methods did not significantly improve immune cell image classification performance”**

We would like to thank all the reviewers again for reading through the manuscript and giving insightful and constructive comments.

We have further refined our manuscript. All changes we made are highlighted in yellow in the revised manuscript.

## Reviewer #1

### Concern #1

I do not find enough merit to support the publication of the paper, as the contribution is not

significant enough. your tests are not sufficient to validate your conclusions. It is not clear the novelty of the method. Are not clear the pros and cons of the proposed approach respect the actual state of the art. Overall impression of study is just below average; the study could be useful and deals with significant problem, but the style way in which paper is written does not match the journal standards.

Thank you for carefully considering the revised version of our manuscript. Our tests were designed according to the purpose of this study, that is to investigate the impact of using different observation methods and settings on cell classification accuracy, and not to exhibit the performance of a novel image analysis method. We agree that this study deals with a significant problem, and believe that many experimentalists and data scientists working in the area of cell imaging would find our results useful in designing and deciding their experiments and data analysis pipelines.

We are thankful to Reviewer #1’s critical readings and comments, which we found very useful. However, some of the comments were a bit abstract and ambiguous, therefore, what the reviewer expects us to improve the quality of the manuscript is not very clear -- (e.g. ‘Overall impression of study is just below average’, ‘the style way in which paper is written does not match the journal standards’). We would very much appreciate it if some more elaboration on the reviewer’s intent and more concrete comments on his/her concerns.

## Reviewer #2

### Concern #1

• Line 94: “three glass-bottom dishes (Eppendorf, Germany). For” – remove the period

• Line 95: “Experiment 1 and 2, respectively.” – Table 1 includes description of Experiment 1. I cannot find anywhere information about Experiment 2. Can you clarify?

Deleted the period in line 94.

The number of cells in experiment 2 is listed in the supplement information. We added that to the caption of table 1.

### Concern #2

Previous comment: “The authors claim that the data are available, but there is no URL pointing to the data.”

o Response: “The data will be made available from the corresponding author upon reasonable request. Since the size of the datasets is larger than 1 TB, it is difficult to put it on a public place with a URL.”

o Recommendation: add a sentence that summarizes the data size reaching 1TB as a multiplication of image pixel size x number of fields of view taken per dish x number of dishes x etc.

A part of the dataset is now available at the following URL.

<https://figshare.com/articles/dataset/Minimum_dataset_for_Different_cell_imaging_methods_did_not_significantly_improve_immune_cell_image_classification_performance_/14789811>

1TB is the total size of the original images. The cropped images were used for training. 0.6% of the original images are now available at the URL above. The cropped images were excluded from the public data because they can be generated from the original images using the method described in the paper.

### Concern #3

Previous comment: “The authors should explain why they used a custom designed CNN architecture as opposed to using a widely used U-Net CNN architecture.

 The authors could include a picture of the custom-designed CNN architecture.

 If the authors are not using the U-Net, then they could compare the CNN architectural designs (custom vs U-Net)”

o Response:” Our CNN network is a plain CNN (no special connections such as skip connections and attention are added), and only changes made were on network parameters such as kernel sizes. Parameters used are given in the section ‘Training and testing of CNN and SVM’

(Main text lines 210-213).

As far as we understand, U-Net is a network for image transformation and is not often used for identification. Although the main focus of this study is to investigate the effect of different observation methods on the accuracy of identification, it is an interesting question whether our findings hold for other architectures (such as ResNet). We have added this point to Discussion (Main text lines 296-302).”

o Recommendation: add a sentence that relates “your definition of a plain CNN” (i.e., there is no published definition of a plain CNN) to a well-known published CNN architecture. For example, you should state how your CNN architecture is different from AlexNet or LeNet and why you made those changes.

We provided the information on the CNN architecture in Main text (lines 212-). As the purpose of our work is not to propose a novel network architecture, we think that our description contains enough information to reproduce our work. However, we added a citation to the paper by Xu *et al* (2017), in which readers can find the exact architecture ours is based on.

## Reviewer #3

### Concern #1

The reviewer's comments were not directly addressed in the revised version of the manuscript. The main claim authors attempting to establish is that regular bright-field images without contrast optics (i.e., phase, DIC) can provide sufficient information for Deep learning. Yet, this was only demonstrated in one biological model system (LMPP vs. proB) with one simple deep learning architecture. Thus, I still don’t think the present data provide sufficient evidence to support the claim.

Thank you for carefully considering the revised version of our manuscript.

*‘The main claim authors attempting to establish is that regular bright-field images without contrast optics (i.e., phase, DIC) can provide sufficient information for Deep learning.’*

-> Yes, it constitutes one of our main claims.

*‘Yet, this was only demonstrated in one biological model system (LMPP vs. proB) with one simple deep learning architecture.’*

-> Yes, it was our experimental design. We found that at least the relatively simple deep learning architecture we used attains acceptable classification accuracy (i.e. AUC >~ 0.9), regardless of some important factors including imaging modalities and focal position,.

*‘Thus, I still don’t think the present data provide sufficient evidence to support the claim.’*

-> We disagree. Our tests were designed according to the purpose of this study, that is to investigate the impact of using different observation methods and settings on cell classification accuracy (and, not to exhibit the performance of a novel image analysis method). We investigated whether it is *possible* to design a simple experimental setup and data analysis pipeline without expensive optics and complicated DL architecture for cell image classification, if such optical setups can provide sufficient information. Logically, this is a yes/no question. Therefore, just one set of clear and statistically sound positive results should convey useful information.

 With that said, we very much agree with the reviewer that more thorough studies should be conducted to further investigate the dependence of the classification performance on different cell types, optics and network architectures. It will be an important follow-up to the current study. In the previous revision, we made this point clear in main text lines 296-303.
