# Supplementary material for: Different cell imaging methods did not significantly improve immune cell image classification performance
Source: PLoS One. 2022 Jan 27;17(1):e0262397. doi: 10.1371/journal.pone.0262397 (PMC8794178; doi:10.1371/journal.pone.0262397)
Supplement: Attachment — Submitted filename: Responses to Reviewers.docx [file pone.0262397.s004.docx]

**Responses to Reviewers’ Concerns for Taisaku Ogawa et al., “Different cell imaging methods did not significantly improve immune cell image classification performance”**

We are thankful to the reviewers for giving useful comments.

Here are our responses to the comments, and all changes we made are highlighted in yellow in the revised manuscript.

Reviewer #2

Concern #1: No further comments raised.

Concern #2

The authors claimed that “A part of the dataset is now available at ..”. However, The URL just shows a list of file folders (dish1, dish3, dish2) and a list of file names. There is no way to download or view the images.

When you open the figshare.com URL below, you see the list of folders and the list of files, but these files are not clickable (weird, but it is how figshare.com founders designed it – probably because they store the data in .zip format only.). Instead, please scroll down and find the red ‘Download’ button next to the ‘Cite’ button, which allows you to download the dataset (3.08GB).

<https://figshare.com/articles/dataset/Minimum_dataset_for_Different_cell_imaging_methods_did_not_significantly_improve_immune_cell_image_classification_performance_/14789811>

Concern #3:

I agree that your description contains enough information about the changes of the UNet architecture. My comment was about explaining why you had to make those changes instead of using the published UNet as is. This comment takes into account your description presented at the lines 211-219:.

Our architecture is identical to Xu’s Deep CNN (we do not use UNet), except that we modified a few parameters regarding the resolution and the number of channels.

We added a sentence to the manuscript to make this point clearer.
